# Identification of metal-centered excited states in Cr(iii) complexes with time-resolved L-edge X-ray spectroscopy†

**DOI:** 10.1039/d4sc07625g

**Published:** 2025-02-26

**Authors:** Nahid Ghodrati, Sebastian Eckert, Mattis Fondell, Andreas Scherz, Alexander Föhlisch, Benjamin E. Van Kuiken

**Affiliations:** a European XFEL 22869 Schenefeld Germany benjamin.van.kuiken@xfel.eu; b Institute for Methods and Instrumentation for Synchrotron Radiation Research, Helmholtz-Zentrum Berlin für Materialen and Energie GmbH 12489 Berlin Germany; c Institut für Physik und Astronomie, Universität Potsdam 14476 Potsdam Germany

## Abstract

New coordination complexes of 3d metals that possess photoactive metal-centered (MC) excited states are promising targets for optical applications and photocatalysis. Ultrafast spectroscopy plays an important role in elucidating the photophysical mechanisms that underlie photochemical activity. However, it can be difficult to assign transient signals to specific electronic excited states and mechanistic information is often inferred from kinetics. Here it is demonstrated that 3d L-edge X-ray absorption spectroscopy is highly selective for MC excited states. This is accomplished by probing the ^2^E spin-flip excited state in Cr(acac)_3_ using synchrotron-based picosecond time-resolved XAS in solution. This excited state of Cr(iii) has the property that its potential is nested with the ground state, which allows for the assessment of purely electronic changes upon excited state formation. Combining the measurements with ligand field and *ab initio* theory shows that the observed spectral changes between the ^4^A_2_ ground state and ^2^E excited state are due to an intensity redistribution among the core-excited multiplets. Extrapolating these results to higher-lying MC excited states predicts that Cr L_3_-edge XAS can distinguish two states separated by ∼0.1 eV despite the L_3_-edge resolution being limited by the 0.27 eV lifetime width of the 2p core-hole. This highlights the potential of L-edge XAS as a sub-natural linewidth probe of electronic state identity.

## Abstract 2

Time-resolved L-edge X-ray absorption spectroscopy is shown to be a state-selective probe by identifying the formation of a spin-flip excited state in Cr(iii).

## Graphical abstract

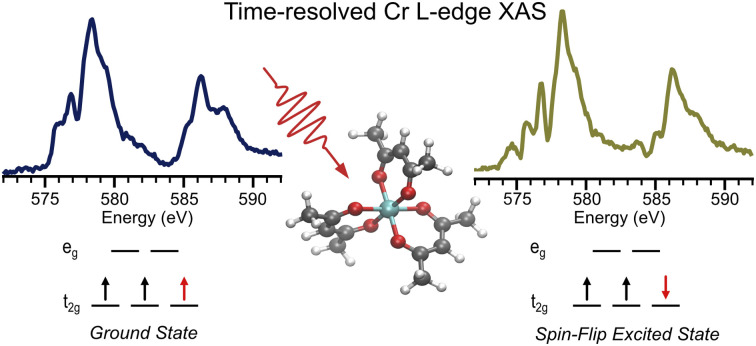

## Introduction

There is much interest in developing photoactive coordination complexes based on earth-abundant 3d transition metals that would supplant the use of precious 4d and 5d elements like Ru, Pt, and Ir.^1,2^ Most of the efforts in this direction have been toward designing complexes that possess long-lived charge transfer (CT) states that mimic the photophysics of their precious metal counterparts. Within this strategy, low-energy metal-centered (MC) excited states are seen as troublesome deactivation pathways, and ligand design seeks to destabilize these states relative to charge transfer excited states.^3–6^ An alternative approach is directly utilizing the MC states in applications. Complexes with emissive MC states may be used for optical devices and sensing applications. In this context, complexes of Cr(iii) exhibiting high quantum yields for spin-flip emission have been of particular interest in recent years.^7–10^ The MC states of 3d complexes are also being increasingly investigated for their bimolecular reactivity, and there have been recent reports of the MC states of Cr(iii), Ni(ii), and Co(iii) complexes performing photocatalysis through either energy or electron transfer mechanisms.^11–16^ As with complexes utilizing CT states, the long lifetime MC states are required, so that photocatalytic activity is not limited by diffusion timescales, and strong-field ligands typically serve to enhance the lifetimes of these states.

**Fig. 1:**
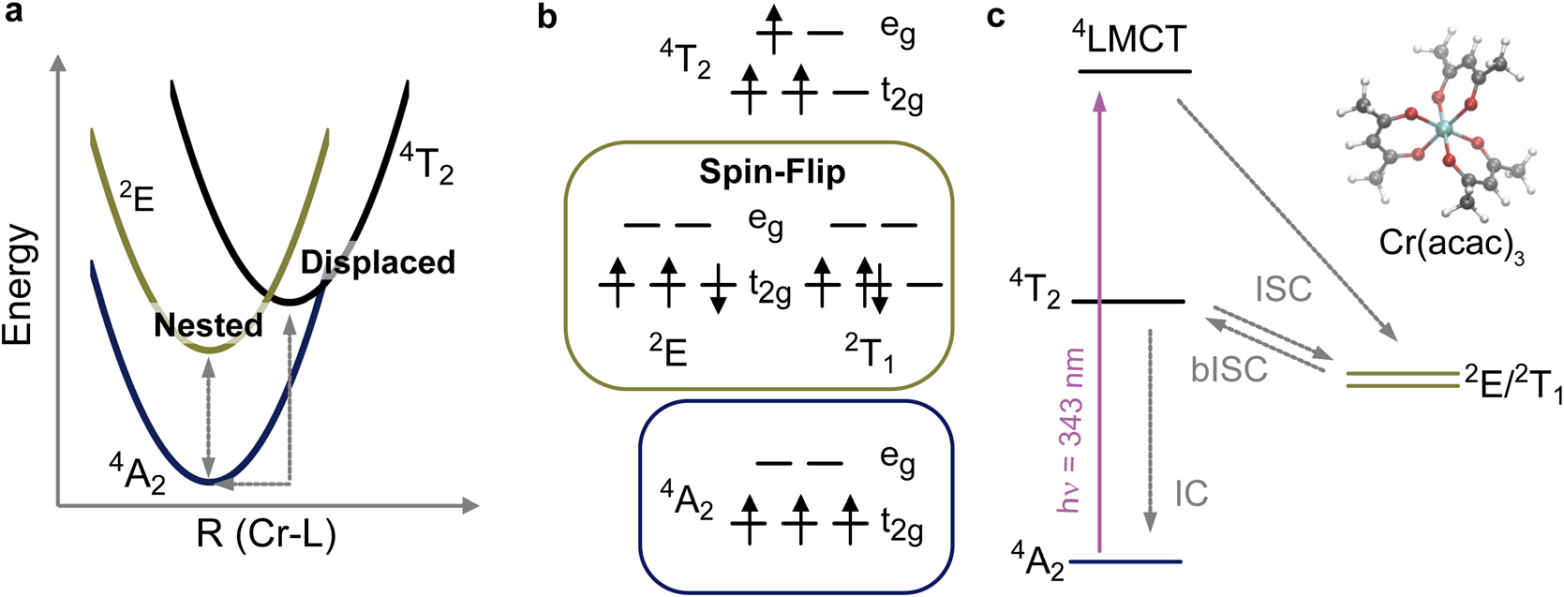
(a) Potential energy curves showing ground and lowest-lying doublet and quartet excited states of a Cr(iii) complex in *O*_h_ symmetry. The intra- (^2^E) and inter-configurational (^4^T_2_) MC excited states give rise to nested and displaced potentials, respectively. (b) Electronic configurations of the low-lying MC states of Cr(iii) complexes. (c) Jablonski diagram of excited state relaxation pathways in Cr(acac)_3_. The non-radiative processes, intersystem crossing (ISC), back-ISC (bISC), and internal conversion (IC) are indicated by grey lines. Vibrational sub-levels are omitted.

Determining the identity and formation pathways of MC excited states can aid in the rational design of new photoactive complexes but can also be experimentally challenging. In electronic spectra, the d–d excitations are often surrounded by charge transfer and/or ligand-based excitations that possess much greater optical strength. Ultrafast visible pump-probe spectroscopy can access MC excited states following their population, but vibronically broadened spectra often do not yield distinct spectral signatures of the electronic excited state, and assignments are often derived from kinetic analysis. Time-resolved L_3,2_-edge X-ray absorption could be an attractive method to provide additional selectivity, as its spectra are comprised of 2p → 3d excitations that directly probe orbitals involved in ligand field photochemistry. In particular, the complexes of the early transition metal ions (*n* = 1–3) give rise to richly featured 2p XAS spectra because the large number of empty 3d acceptor orbitals yields well-resolved 2p–3d exchange-split multiplets.^17–20^ This suggests that it could be a sensitive probe, but to the best of our knowledge, there are no previous examples of time-resolved L-edge XAS for 3d^*n*^ complexes with *n* < 5. There are numerous studies that have utilized pico- and femtosecond L_3,2_-edge XAS in solution to study photoinduced spin crossover, charge transfer, photodissociation, and photosolvation processes,^21–28^ but each of these cases involves a large geometric perturbation or a change in the d-electron count. Alternatively, the pseudo-octahedral complexes of Cr(iii) provide minimally perturbed excited states because photoexcitation from the ^4^A_2_ ground state is followed by intersystem crossing (ISC) that leads to the formation of an intraconfigurational ^2^E spin-flip state (see Fig. 1a and b). The ^4^A_2_ ground state as well as the lowest lying doublet states, ^2^E and ^2^T_1_, arise from (t_2g_)^3^ electron configurations as indicated in the diagrams in Fig. 1b. The ground and excited state potentials are nested due to the conserved electron configuration.^29,30^ Here we employ picosecond time-resolved L-edge XAS to observe the L-edge signature of the ^2^E spin-flip excited state in Cr(acac)_3_.

Cr(acac)_3_ has served as a model for ultrafast spectroscopic studies of the photophysics of Cr(iii) complexes. A schematic of its photophysics accompanying LMCT excitation is shown in Fig. 1c. The state diagram has been simplified to *O*_h_ (rather than *D*_3_) symmetry and vibrational sub-levels of the states have been omitted. McCusker and coworkers identified the initial ISC time following CT excitation and ^2^E formation within ∼100 fs and discussed the role of coherent nuclear dynamics in the process.^31–33^ Maçôas *et al.* used transient infrared (IR) spectroscopy to track vibrational energy relaxation providing a detailed kinetic model of the photophysics.^34,35^ This model quantified the competition between vibrational cooling of the ^2^E state (∼7 ps) and thermally activated back-ISC (bISC), which leads to ground state recovery *via* internal conversion. By ∼15 ps thermalized populations of ^4^A_2_ and ^2^E are found, and the doublet yield is between 15 and 30% depending on pump wavelength. Both methods show the remaining ^2^E population returns to the ground state non-radiatively with a time constant of ∼800 ps. It is notable that the spectroscopic signature of the bISC process is not clear in either measurement. A 1.56 ps time constant was determined for the internal conversion ^4^T_2_ → ^4^A_2_, but no signature of the ^4^T_2_ state was observed despite the kinetic model predicting a significant ^4^T_2_ population persisting on this timescale.^35^ Similarly, the optical measurements identify a ∼1 ps time constant associated with band narrowing of the excited state absorption but no distinct signature of a second excited state. Together these examples illustrate how a complimentary probe of electronic state identity would aid in elucidating fundamental photophysical mechanisms.

First, we present the changes in the Cr 2p XAS spectrum upon excitation of its LMCT band, and kinetic analysis suggests that the difference spectrum measured at 75 ps is the signature of the ^2^E spin-flip state. This assignment is confirmed spectrally by comparison with both *ab initio* quantum chemical and ligand field theoretical calculations. Analysis of the calculated spectra in terms of orbital and spin contributions reveals how spectral intensity is redistributed among the core-excited states when a new valence state forms. Finally, computationally extrapolating to higher-lying excited states arising from the ^2^T_1_ term shows that Cr L_3_-edge yields a state-selective probe with sub-natural linewidth sensitivity. These results suggest that L-edge XAS can effectively track electronic population redistribution in cases where other spectroscopies yield ambiguous signatures.

## Results and discussion

### Time-resolved Cr L-edge XAS of Cr(acac)_3_

**Fig. 2:**
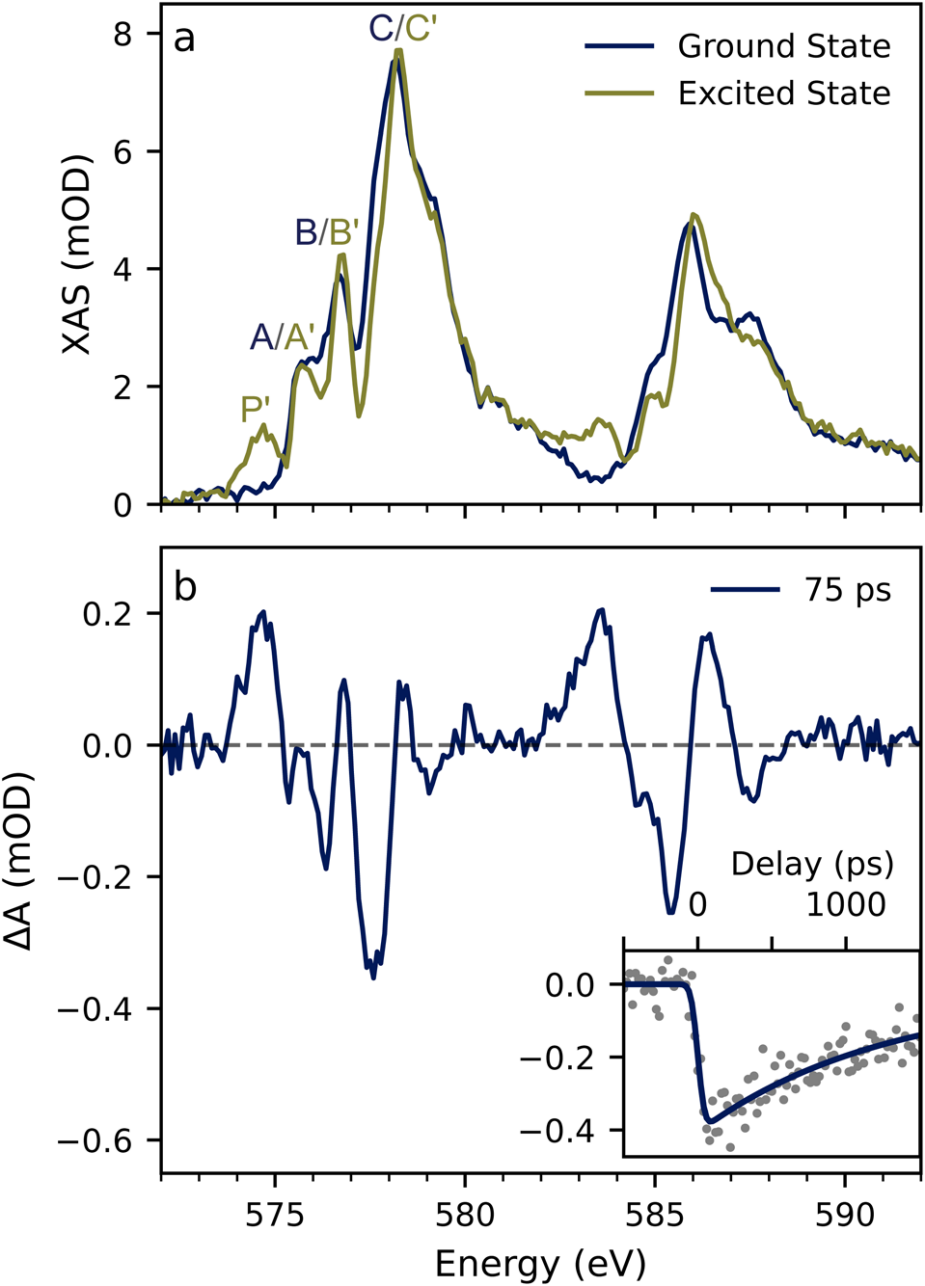
Steady-state and transient L-edge XAS spectra of Cr(acac)_3_. (a) The solution phase L_3,2_-edge XAS spectrum for the ground state and excited state at 75 ps after excitation. The excited state spectrum is generated using an excitation fraction of 20%. Features are labeled A, B, and C for the ground state, and (P′), (A′), (B′), and (C′) for the excited state. (b) The transient Cr L-edge XAS spectrum was measured at a 75 ps delay following 343 nm excitation. The inset shows the time trace taken at 577.6 eV together with the single exponential fit of data (*τ* = 1.26 ns).

The transmission mode Cr 2p XAS spectrum of a 15 mM Cr(acac)_3_ dissolved in a 90 : 10 EtOH : DMSO mixture is shown in Fig. 2a for both the ground and excited state. The ground state spectrum shows three prominent features in the L_3_-edge (2p_3/2_ → 3d) at energies of 575.7, 576.7, and 578.2 eV denoted A, B, and C in Table 1 together with assignments which are explained below. The L_2_-edge (2p_1/2_ → 3d) is found between 585 and 589 eV with the intensity maximum at 585.9 eV and possesses a somewhat different lineshape than the L_3_-edge. This spectrum agrees well with those previously reported for solid-state (powder) and solution samples.^19,20,36^

**Table 1:** Energies and assignments of each major spectral feature observed in the L_3_-edge XAS spectra for the ^4^A_2_ ground state and ^2^E excited state

Label	Energy (eV)	Assignment
A	575.7	2p → t_2g_, 2p → e_g_ (Δ*S* = 0)
B	576.7	2p → e_g_, (Δ*S* = 0, ±1)
C	578.2	2p → e_g_ + t_2g_ → e_g_ (Δ*S* = 0, ±1)
P′	574.6	2p → t_2g_, 2p → e_g_ (Δ*S* = 0, +1)
A′	575.7	2p → e_g_ (Δ*S* = 0, +1)
B′	576.8	2p → e_g_ (Δ*S* = 0, +1)
C′	578.3	2p → e_g_ + t_2g_ → e_g_ (Δ*S* = 0, +1)

The XAS difference spectrum measured at 75 ps following 343 nm excitation of the Cr(acac)_3_ LMCT band is shown in Fig. 2b. The transient spectrum exhibits many extrema owing to the rich multiplet structure of the Cr L-edge. There is a loss of intensity at 577.7 eV on the low-energy side of the C feature with a slight increase at 578.3 eV. There is an overall loss of intensity in the region of the A and B features. Most notably, there is a clear excited state absorption feature at 574.5 eV, which appears below the ground state L_3_-edge. The changes at the L_3_-edge are mimicked at the L_2_ with the increase of intensity on the low-energy side of the edge at 583.5 eV and a decrease at 585.9 eV.

The spectrum of the excited state at 75 ps delay is shown in Fig. 2a. This spectrum has been constructed from that of the ground state and the difference spectrum assuming a 20% excitation yield. This choice is rationalized by comparing spectra constructed with a series of excitation fractions (see Fig. S4† and the associated discussion). The excited state spectrum exhibits four features tabulated in Table 1. Three of which appear at nearly the same energies as the ground state features and are denoted A′, B′, and C′ with energies at 575.7, 576.8, and 578.3 eV, respectively. The fourth feature is a distinct pre-edge peak at 574.6 eV denoted P′. In this work we use the term pre-edge to mean a feature that appears energetically below the ground state absorption. As with the ground state, the changes are mimicked at the L_2_-edge. There is a slight shift to the higher energy of the most intense feature (∼586 eV) and the appearance of a corresponding pre-edge peak in the middle of the L_3_–L_2_ gap at 583.5 eV.

The inset in Fig. 2b shows the time dependence of the transient signal measured near the maximum amplitude of the loss feature at the L_3_-edge (577.8 eV). The transient signal forms within the ∼70 ps time resolution of the experiment and yields a time constant for ground state recovery of ∼1.26 ns. The single exponential kinetics are in agreement with the expectation that the difference spectrum at 75 ps is due to the ^2^E excited state, but this value is somewhat longer than previous studies that found a ∼800 ps ^2^E lifetime for room temperature measurements.^31,34,35^ The longer lifetime observed in the X-ray measurement is likely due to a lower sample temperature. Cr(acac)_3_ has a millisecond lifetime under cryogenic conditions, and the kinetic model derived from transient IR spectroscopy found a 25 kJ mol^−1^ activation barrier for ^2^E → ^4^T_2_ bISC process.^35^ With this activation barrier, a 0 °C sample solution would already result in a longer lifetime by a factor of two. While the exact sample temperature is unknown, rapid evaporative cooling of EtOH flat jets in vacuum (∼10^−3^ mbar) can result in sample temperatures as low as −20 °C rationalizing the longer lifetime,^37^ and we assign the measured difference spectrum to the ^2^E state as expected.

### Origin of ground and excited state spectral features

We now consider how the identity of the electronic states of Cr(acac)_3_ is encoded in the 2p XAS. Ignoring any CT effects, the L-edge spectrum of Cr(iii) arises from excitations between all populated valence states and the 1260 2p^5^3d^4^ core-excited states. Here we employ both quantum chemical and model Hamiltonian approaches. Firstly, ligand field multiplet theory (LFMT) is used with previously published parameters from optical spectroscopy.^38,39^ Secondly, complete active space configuration interaction (CASCI) calculations are carried out to calculate the spectra from molecular orbital calculations.^36^ The active space for the CASCI calculation includes the Cr 3d and 2p orbitals. Thus, both approaches solve a full CI problem for a configuration space that includes all 2p and 3d electrons and orbitals. In the LFM simulation, a basis of spherical harmonics is used and the Hamiltonian is empirically parameterized. In CASCI calculation, the interactions are calculated *via* quantum chemical methods and the calculations include the complete electronic system. We focus our analysis on the L_3_ spectra, since the L_2_ spectra mirror the L_3_ but are further broadened by additional Auger (Coster-Krönig) relaxation channels.

**Fig. 3:**
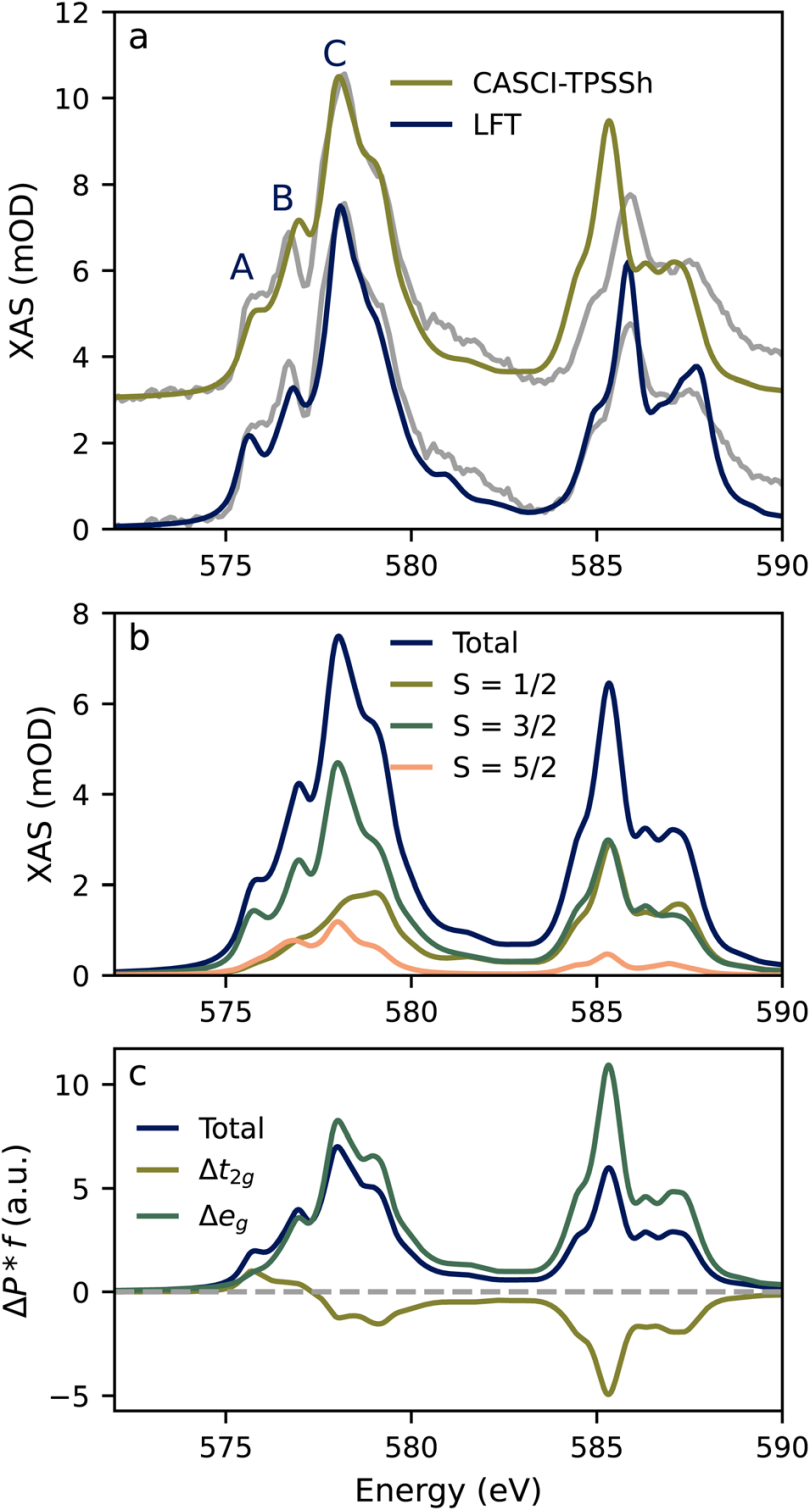
Ground state spectrum. (a) Comparison of experimental and calculated Cr 2p XAS spectra for the ^4^A_2_ ground state of Cr(acac)_3_. Experimental spectra (gray) are overlayed with spectra generated from LFT and CASCI using orbitals from a TPSSh DFT calculation. (b) Fractional spin decomposition for the spectrum calculated at the CASCI-TPSSh level. The total spectrum (shown in blue) is the sum of the individual spin state contributions. (c) Intensity-weighted (*f* = oscillator strength) Löwdin orbital population difference (Δ*P*) spectra show the change between the ground state (t_2g_)^3^ occupancy and the d-orbital occupancy of the core-excited state.

The experimental ^4^A_2_ ground state L-edge spectrum is shown together with the calculated spectra using both LFMT and CASCI in Fig. 3a. Firstly, the LFMT simulation (blue, bottom) gives excellent agreement with the experimental spectrum. The ligand field parameters were taken to be the optically determined values reported in the work of Atanasov and Schönherr with 10 Dq, B and C equal to 2.32 (18 700), 0.06 (500), and 0.42 (3400) eV (cm^−1^), respectively.^40^ These Racah parameter values correspond to a ∼50% reduction of 2-electron integrals from atomic references and a C/B ratio of 6.8 compared to the nominal value of ∼4. A full set of parameters is given in Table S1.† These values are consistent with a significant nephelauxetic reduction and metal–ligand covalency. A similar reduction of the 2-electron interactions and large C/B ratio was recently utilized to interpret the X-ray and optical data of the Cr(iii) sites in ruby.^41^

Next, the CASCI spectrum (Fig. 3a, top) also gives good agreement with both the experiment and the LFT simulation for L_3_-edge. The only discrepancy is an overestimation (∼0.2 eV) of the energy of the B feature. The L_2_-edge is also well-reproduced, but the CASCI approach underestimates the L_3_–L_2_ splitting, which is common for approaches that employ perturbative treatments of SOC. This has been previously discussed for *ab initio* calculations of L-edge spectra.^17,42–44^ The CASCI result is denoted CASCI-TPSSh because the calculation employs quasi-restricted orbitals (QROs) from a TPSSh DFT calculation for the orbital basis in the CI calculation. DFT orbitals have been successfully combined with CI-based methods for calculating various L- and K-edge X-ray spectroscopies.^39,42,45–47^ In the case of Cr(acac)_3_, we found a direct relationship between the fraction of Hartree–Fock (HF) exchange in the DFT functional and the energetic spread of the multiplets in the XAS spectra (see Fig. S7 and S8 of the ESI†). Employing a functional with a high HF exchange fraction leads to broader L-edges. The QROs derived from the TPSSh functional with 10% HF exchange were found to give the best agreement between theory and experiment. We stress that we do not consider this approach to be a general predictive *ab initio* method, and the combination of DFT and CI results in double-counting of Coulomb interactions. Rather, in the case of Cr(acac)_3_, the use of TPSSh QROs reproduces the ground state experimental spectrum and spectrum predicted by LFMT. The CASCI method gives a set of orbitals rather than a set of parameters, includes the true molecular symmetry, and offers the possibility of using the standard tools of quantum chemistry to analyze the result. Further, as discussed in the ESI (Fig. S9†), purely *ab initio* CASCI calculations based on CASSCF orbitals were not able to satisfactorily reproduce the ground state spectrum. This is consistent with previously reported *ab initio* calculations of the Cr(acac)_3_ L_3_-edge where the intensity ratio between A and B is either underestimated,^20^ or the A feature is nearly missing.^36,48^

The spin state and orbital contributions to the Cr 2p XAS spectrum for the CASCI method are shown in Fig. 3b and c, respectively. It shows that the intensity of the L_3_ spectrum is dominated by quartet transitions with doublet and sextets contributing 26% and 14%, respectively. The L_2_-edge has near equal contributions from quartets and doublets with only a minor intensity due to sextets. The A feature has a large quartet character while the doublet and sextet contributions gain intensity for B and C. This spin state distribution agrees well with that obtained from LFMT (Fig. S6†) further emphasizing the correspondence between ligand field theory and the quantum chemical approach. We continue using the CASCI results throughout the rest of the discussion.

The orbital contributions in Fig. 3c are assessed by constructing intensity-weighted orbital difference plots that track the change in the populations of the 3d orbitals following core excitation. It can be seen that the lower-energy excitations comprising the A feature have significant 2p → t_2g_ character with a similar fraction of 2p → e_g_. The B feature is almost purely 2p → e_g_. The states contributing to C have a negative t_2g_ fraction signifying a significant double-excitation character. This double excitation character arises when core excitations couple with valence t_2g_ → e_g_ transitions. The combination of spin and orbital decompositions allows us the make the qualitative assignments of the A, B, and C features given in Table 1.

**Fig. 4:**
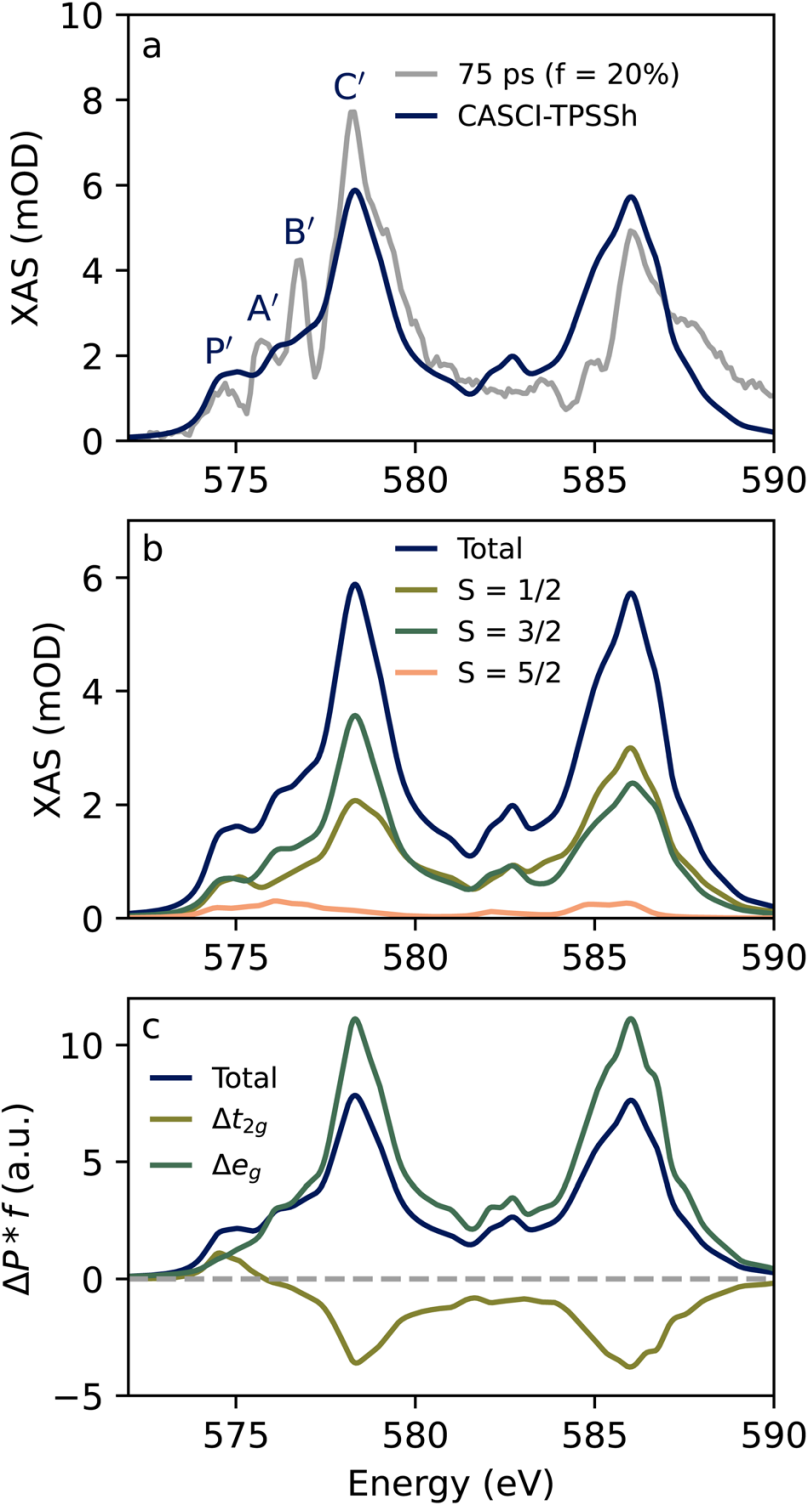
Spin-flip excited state spectrum. (a) Comparison of the experimental excited state (gray) assuming a 20% excited state fraction and calculated Cr 2p XAS Spectra for the ^2^E state of Cr(acac)_3_. (b) Fractional spin decomposition for the spectrum calculated at the CASCI-TPSSh level. The total spectrum (shown in blue) is the sum of the individual spin state contributions. (c) Intensity-weighted Löwdin orbital population difference spectra show the change in the ^2^E state (t_2g_)^3^ occupancy and the d-orbital occupancy of the core-excited state.

Fig. 4a shows the comparison of the excited state spectrum with the spectrum of the lowest energy doublet state (^2^E) calculated at the CASCI level of theory. For the calculation, we utilize the fact that the ^2^E state possesses a nested potential as described above.^30^ Thus, the same geometry is used as for the ground state, and in the CASCI calculations the metal-centered excited states are constructed from the same orbitals as the ground state. The calculated spectrum captures all major features of the experimental spectrum though agreement is not as high as the ground state. The A′ and B′ features of the spectrum are significantly sharper than the features in the calculated spectrum. These discrepancies could be a manifestation of the limited accuracy of the methods employed because it has been shown that correlated calculations with large active spaces can be necessary to accurately reproduce the L-edge spectra.^43^ Alternatively, some geometric relaxation of the excited state might require a unique set of orbitals (and ligand field parameters) to more accurately describe the ^2^E spectrum. That said, a relaxed ^2^E structure produced by a broken-symmetry DFT optimization exhibits changes of the Cr–O bond distances of only ∼1 pm suggesting that the nested potential approximation is valid. Despite the differences between theory an experiment, the calculation contains the P′ feature that is unique to the excited state, and there is little shift in the maximum of the L_3_-edge upon ^2^E formation as in the experiment. Fig. 4b shows the spin state decomposition analysis of the ^2^E spectrum. Doublets and quartets make nearly equal contributions across the spectrum except for the B′ and C′ regions where the quartet weight is ∼50–100% higher than the doublet. The sextet states on the other hand make almost no contribution to the spectrum. This can be explained because Δ*S* = 2 transitions are not directly enabled by SOC. The SOC operator only connects states with Δ*S* = ±1, and the small sextet intensity could arise, for example, due to the fact that the ^2^E state already includes some quartet character due to 3d SOC. The orbital decomposition in Fig. 4c gives an interpretation that is similar to the ground state. The low, intermediate, and higher energy features have transition character of 2p → t_2g_, 2p → e_g_, and double excitation, respectively yielding the qualitative assignments in the right column of Table 1.

**Fig. 5:**
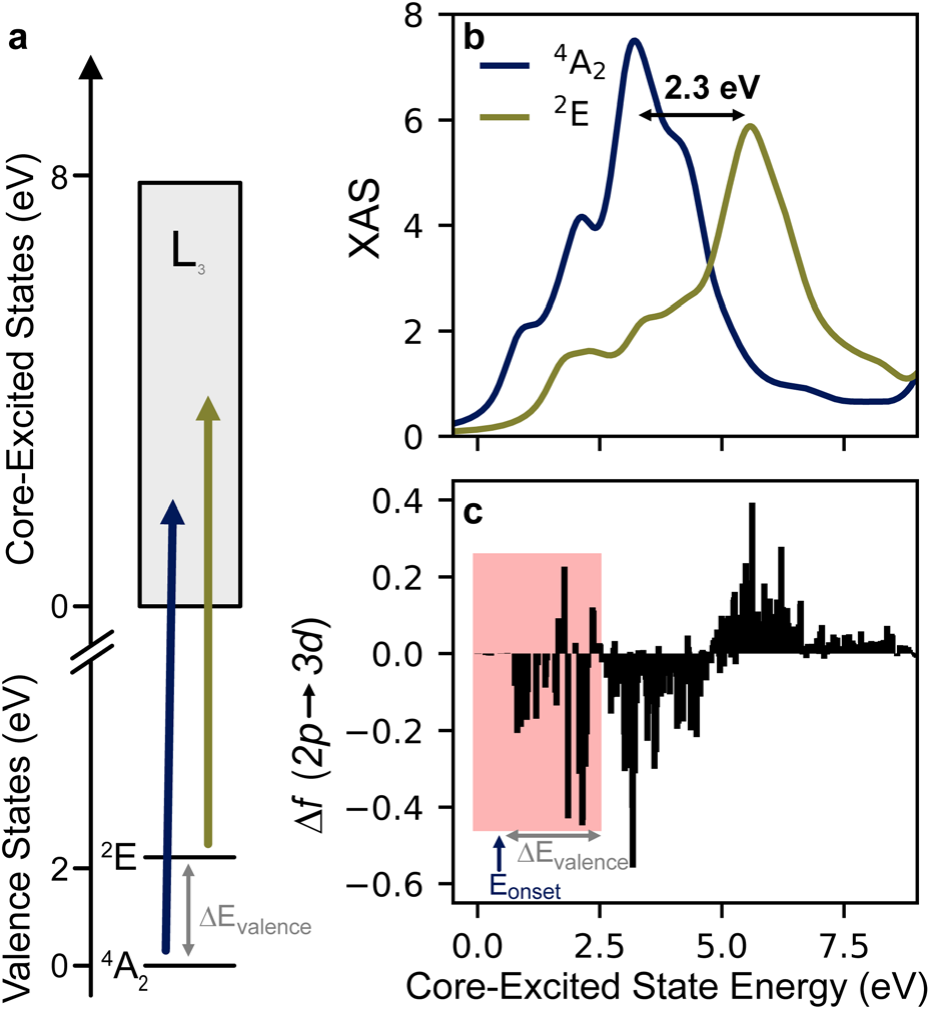
Core-excitation processes for two valence states with nested potentials. (a) Schematic of XAS process for ^4^A_2_ and ^2^E showing the 2.05 eV separation predicted at the CASCI-TPSSh level of theory. (b) XAS spectra are plotted on the core excited state energy axis, where the first core excited state is taken to be 0 eV. (c) Oscillator strength differences (Δ*f*_2p→3d_ = *f*_2p→3d_(^2^E) − *f*_2p→3d_(^4^A_2_)) for each core-excited state. The pink shaded region indicates the core-excited states that can give rise to spectral features (here P′) that appear below the onset of the absorption (*E*_onset_) of the ^4^A_2_ ground state.

For further insight into the L-edge intensity differences between two MC states, we consider how the core and valence excited states relate to each other. Fig. 5a depicts core-excitation process giving rise to the L_3_-edge for both the ^4^A_2_ and ^2^E states Cr(acac)_3_. The manifold of core-excited states is the same regardless of whether the system is in the ground or valence excited state. This is not generally the case, but it is due to the nested potentials of the ^4^A_2_ and ^2^E states as described above. If the transition strengths between core and valence states were the same regardless of which MC state is populated, the 2p XAS spectrum would red shift by an energy equal to the gap between valence states. This is clearly not the case as the appearance of the P′ feature is a clear change in the spectral profile (see Fig. 3a and 4a), and the intensity maximum (C/C′) only changes by ∼0.1 eV experimentally or ∼0.24 eV for CASCI-TPSSh. These observations are evidence of a redistribution of intensity amongst the 2p^5^3d^4^ multiplets. This effect can be more clearly seen by viewing the theoretical spectra on the energy axis of core-excited states, which displays the final states contributing to the observed spectrum. This axis is defined by setting the energy of the first 2p core-hole state to 0 eV, which happens to be a sextet state with 2p^5^(t_2g_)^3^(e_g_)^1^ character that gives no intensity to either the ground or excited state. The spectral profiles on this axis are shown in Fig. 5b, and the change in the oscillator strength (Δ_2p→3d_) associated with ^2^E formation is shown in Fig. 5c for each 2p^5^3d^4^ state.

The shift of intensity to higher energy core-excited states upon ^2^E formation is clearly seen in Fig. 5b. The spectral profile shifts by 2.3 eV to higher energy, and the Δ*f* spectrum (Fig. 5c) shows a dispersive shape with negative and positive features dominating below and above 5 eV, respectively. This energy shift is approximately the energy of the valence excited state (2.05 eV at the CASCI + TPSSh level) plus the calculated peak shift of 0.24 eV. Thus, we conclude that there is a significant additive component to how intensity of the 2p XAS is shifted upon formation of the MC excited state. Many core-excited states that give intensity to the XAS of an MC excited state are separated by the valence excitation energy from the states that give intensity to the ground state. This accounts for the fact that the A′, B′, and C′ features do not shift relative to their ground state counterparts.

Focusing now on the low-energy region, it can be seen that the core-excited states that give rise to the A feature do not contribute to the ^2^E spectrum. The Δ*f* values around ∼1.0 eV are negative, and the P′ is due to transitions at 1.7 and 2.4 eV, which still appear below the A feature on the spectroscopic (measurement) axis (Fig. 2a). By energy conservation, there will be a set of core excited states that can only contribute to the pre-edge region of the spectrum of a valence excited state. For the ^2^E state these core-excited states are shown in the shaded region of Fig. 5c. The upper boundary of this set is given by the sum of the onset of ground state absorption (*E*_onset_ ≈ 0.5 eV) plus the valence excitation energy (Δ*E*_valence_ = 2.05 eV). As the valence excitation energy increases, more core excited states enter the shaded region where they can give intensity to the pre-edge region that does not overlap with ground state absorption. This makes this low-energy region of the 2p XAS particularly sensitive to the identity of MC excited states. This is further explored computationally for the states arising from the ^2^T_1_ term in the next section.

### Sub-natural linewidth state selectivity

**Fig. 6:**
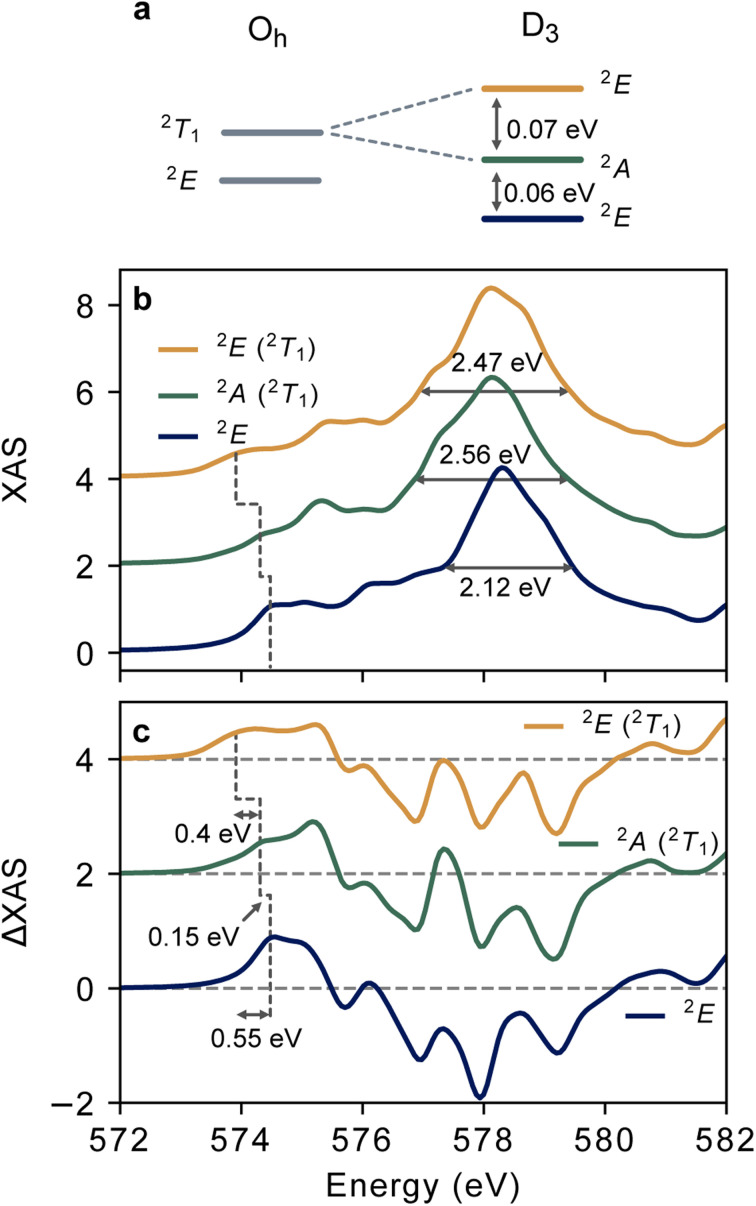
Calculated L_3_ XAS of the ^2^E/^2^T_1_ manifold. (a) Depiction of descent in symmetry from *O*_h_ to the *D*_3_ point group of Cr(acac)_3_ together with energy splittings between doublet states predicted by CASCI-TPSSh. (b) L_3_-edge XAS of the lowest-lying doublet states of Cr(acac)_3_ calculated at CASCI-TPSSh level of theory. Horizontal arrows indicate the FWHM of the most intense feature. (c) Theoretical difference spectra for each doublet state from the ground state L_3_-edge. Vertical elbows schematically indicate the shift of pre-edge absorption onset.

Fig. 6 shows the manifold of low-lying doublet states considering the actual *D*_3_ symmetry of Cr(acac)_3_. The ^2^T_1_ is split into ^2^A_2_ and ^2^E terms as depicted in Fig. 6a. The CASSCI-TPSSh energy gap between the ^2^A_2_ (^2^T_1_) and ^2^E is 0.06 eV, and the ^2^A_2_ (^2^T_1_) state is separated by 0.07 eV from the ^2^E (^2^T_1_) term. We note that the CASCI-TPSSh calculation yields excitation energies for this doublet manifold that are nearly identical to those calculated by CASSCF(13,13) + NEVPT2 as described in the ESI.† Overestimation of spin-flip excitation energies in Cr complexes is common even for high-level quantum chemical calculations.^8,49,50^ However, the energetic spread of the doublet band is consistent with that of the low-temperature absorption spectrum which spans 1.6 to 1.8 eV.^51^ We note that for the vibrationally relaxed sample of Cr(acac)_3_ (flowing in a cold in-vacuum liquid jet), the Boltzmann population of the lowest energy (^2^E) state is estimated to be ∼95%.

The calculated Cr L_3_-edge XAS of the ^2^E, ^2^A_2_ (^2^T_1_), and ^2^E (^2^T_1_) states are shown in Fig. 6b. There are clear differences in the calculated XAS of these three electronic states, which are annotated for the shift of the lowest energy feature and change of FWHM for the most intense feature. Due to the magnitude of the discrepancies between the calculated and experimental ^2^E spectrum discussed above, it would be difficult to distinguish *a priori* which of these doublets is observed if it were not known that the ^2^E dominates the spectral weight. The similarity of the calculated doublet spectra to each other suggests that systematic errors in the calculations are conserved across this manifold of states. Thus, we compare the differences between the computed spectra to characterize the magnitude of spectral changes one might expect for such a set of MC excited states. For a clearer comparison of these changes, the calculated difference spectra between the doublet states and the ground state are shown in Fig. 6c.

First, the FWHM of the most intense feature increases by ∼0.4 eV on going from the ^2^E state to either of the higher energy states. This change is manifested in the difference spectrum in the significant increase in ΔXAS around 577.5 eV relative to the ^2^E difference spectrum. While changes in this region contain several overlapping contributions between the ground and excited states, the pre-edge peak P′ is an isolated signature. This difference feature shifts by 0.15 eV to lower energy on going from the ^2^E to the ^2^A_2_ state with an additional 0.4 eV shift to lower energy upon the formation of the ^2^E (^2^T_1_). This result suggests that larger spectral shifts may be observed than the energy differences between the states. The 0.55 eV shift of the P′ feature between the ^2^E and the ^2^E (^2^T_1_) states is more than four times greater than the 0.13 eV energy gap, and twice as large as the 0.27 eV core-hole lifetime broadening of the Cr L_3_-edge. This can be rationalized by the 2p–3d exchange which results in a large energetic spread of the core-excited multiplets enhancing the spectral contrast between valence states of similar energy and character. Thus, we see that the pre-edge region identified in the previous sections is indeed predicted to contain state-selective signatures.

The differences described above should be observable given a sufficient excited state population. Any contribution from the ^2^T_1_ term would be well within the error of calculated spectra for the present case. At earlier delay times there could be much more significant populations of higher energy MC states. Firstly, bISC will give rise to a ^4^T_2_ population prior to the ∼1 ps internal conversion timescale as described in the introduction. Second, LMCT excitation is expected to yield an internal temperature of 667 K,^35^ and vibrational energy relaxation could be accompanied by a redistribution of electronic population among the doublet states, which would be manifested in a time-dependent shift of the pre-edge peak. This could have important implications for understanding dynamics because the ^2^T_1_ states are expected to be more strongly distorted from the ground state geometry than the ^2^E state.^29^ It was recently suggested by Heinze and coworkers that a distorted geometry of the ^2^T_1_ states in the molecular ruby complex [Cr(tpe)_2_]^3+^ could serve to accelerate ground state recovery in a system where bISC is energetically unfavorable.^13^ Probing these phenomena would require sub-ps time resolution, which is routinely achievable for the L-edges of 3d transition metals at X-ray free electron laser (FEL) facilities. The development of high-sensitivity setups for soft X-ray absorption spectroscopy at FELs is an ongoing area of development,^52^ and liquid jet sample environments are being provided at both the European XFEL and the Linear Coherent Light Source (LCLS) at Stanford.

## Conclusions

In summary, the electronic state selectivity of 3d L-edge XAS has been assessed by probing the spin-flip excited state in Cr(iii). Theory predicts that this sensitivity is not limited to the lowest lying levels. Each MC excited state is likely to give rise to a unique spectral profile. For spin-flip systems, the sensitivity of the background-free low-energy region of the spectrum to the identity of MC excited states is expected to be a general feature, and the methods used here could readily be extended to other d-block elements that form complexes with spin-flip excited states. Although we have used the nested potentials of spin-flip states to simplify our analysis, the sensitivity of 2p XAS to MC state identity is not limited to this case. For example, the MC excited states of Fe and Ni complexes exhibiting large geometric distortions from their ground states have been characterized by 2p XAS.^21,27^ The sensitivity to the identity of MC excited states stands alongside the already established sensitivity of 2p XAS to changes in oxidation state and molecular geometry. Ongoing technical developments in both theory and experiment are poised to enable 2p XAS to address photochemical questions in a broader range of systems. L-edge XAS of 3d complexes does not rely on being able to distinguish states on the valence state energy scale. Instead, the core-excited states act as spectators, and two nearly degenerate valence states can possess significantly different L-edge multiplets.

## Methods

### Experimental details

Cr(acac)_3_ was purchased from Merck (Sigma Aldrich). Samples were prepared at 15–20 mM concentrations and filtered before introduction to the chamber. Samples were kept out of direct light during measurement and UV/vis showed no signs of sample degradation over the course of the experiment.

The solution phase L-edge XAS experiments were performed at the AXSYS-NEXAFS endstation at the UE52-SGM beamline of Bessy II.^53^ A colliding nozzle flat jet delivered samples to the interaction region (Advanced Microfluidic Systems GmbH). The sample solution and transfer lines were cooled to decrease the vapor pressure of the sample solution increasing liquid jet stability. The liquid jet utilized 2 × 20 μm nozzles with a total flow rate of ∼1 ml min^−1^. It is noted that the addition of 10% DMSO to the ethanol solvent led to a significant stabilization of the in-vacuum liquid jet and improved S/N. Optical pump picosecond soft X-ray probe measurements were carried out utilizing the hybrid operation mode of the BESSY-II storage ring. In this mode, a temporally isolated “CAMSHAFT” X-ray pulse is used as the probe. The XAS signal was collected by measuring the transmitted X-ray intensity on an APD. The APD signal was fed into a lock-in amplifier to isolate the CAMSHAFT signal. The X-ray pulse was spatially (on a YAG screen) and temporally overlapped with a UV pump pulse to excite the sample. The initial time-zero determination was performed using the N K-edge signal of Fe(bpy)_3_Cl_2_. The UV pulse was taken as the 3rd harmonic of 1030 nm fundamental of a Pharos (Light Conversion) laser operating at 208.3 kHz. The laser fluence on the jet was ≈50 mJ cm^−2^. Further details related to O K-edge background subtraction, solvent dependence of the signal, and kinetic modeling can be found in the SI.

### Computational details

LFMT simulations were performed utilizing a model Hamiltonian which includes two-electron interactions (*Ĥ*_ee_), SOC (*Ĥ*_SO_), and an octahedral crystal field (*Ĥ*_CF_). The two-electron interactions were expressed in terms of the Slater–Condon parameters. The simulations were performed using the EDRIXS Python modules to calculate all matrix elements.^54^ A complete set of ligand field parameters can be found in the SI.

All quantum chemistry calculations were performed with Orca (Version 5.0.4).^55^ The def2-TZVP basis set was used.^56^ The conductor-like polarizable continuum model was used in all calculations with the default parameters for ethanol.^57^ The ground state structure of Cr(acac)_3_ was optimized using the BP86 functional.^58,59^ The D3BJ dispersion correction was applied.^60^ CASCI calculations followed the protocol of Chantzis *et al.* for L-edge XAS spectra, which utilizes an active space of the five Cr 3d orbitals and the three Cr 2p orbitals.^36^ All roots corresponding to valence and singly core-excited states were calculated. This means 15 sextet, 160 quartet, and 325 doublet states. The RI approximation was used in the integral transformation step and the def2-TZVP/C basis set was used.^61^ SOC was then included by the spin–orbit mean-field approximation.^62^ Spectra for each multiplet (^4^A_2_, ^2^E, *etc.*) were computed using an equal weighting of each microstate within the term. The transition dipole moments for valence-excited state spectra were requested by computing an increased number of initial states in the SOC step of the calculation. Details of spin and orbital decompositions are left to the ESI.†

For each method, the calculated spectra have been shifted to align their maxima with the C feature in the experimental spectrum of the ground state. The same energy shift is applied to the calculated excited state spectra.

## Data availability

The data supporting this article have been included as part of the ESI.† Additional data are available from the corresponding author upon request.

## Author contributions

B. V. K. conceptualized the research. S. E., M. F., and B. V. K. carried out the experiment. S. E. and B. V. K. analyzed the experimental data. N. G. and B. V. K performed the calculations and wrote the initial draft of the manuscript. N. G., S. E., M. F., A. S., A. F., and B. V. K. reviewed and edited the manuscript.

## Conflicts of interest

There are no conflicts to declare.

## Supplementary Material

SC-OLF-D4SC07625G-s001
