# Supplementary material for: Identification of metal-centered excited states in Cr(iii) complexes with time-resolved L-edge X-ray spectroscopy
Source: Chem Sci. 2025 Feb 26;16(15):6307–16. doi: 10.1039/d4sc07625g (PMC11895842; doi:10.1039/d4sc07625g)
Supplement: SC-016-D4SC07625G-s001 [file SC-016-D4SC07625G-s001.pdf]

Supplementary Information (SI) for Chemical Science.
This journal is © The Royal Society of Chemistry 2025

Supporting Information: Identification of Metal-Centered Excited States in Cr(III) Complexes with Time-Resolved L-edge X-ray Spectroscopy

Nahid Ghodrati,[†] Sebastian Eckert,[‡] Mattis Fondell,[‡] Andreas Scherz,[†] Alexander Föhlisch,[‡,¶] and Benjamin E. Van Kuiken[*,†]

†*European XFEL, 22869 Schenefeld, Germany*

‡*Institute Methods and Instrumentation for Synchrotron Radiation Research, Helmholtz Zentrum Berlin für Materialen and Energie GmbH, 12489 Berlin, Germany*

¶*Institut für Physik und Astronomie, Universität Potsdam, 14476 Potsdam, Germany*

E-mail: benjamin.van.kuiken@xfel.eu

Data Processing and Analysis

Solvent Background Subtraction

The transient difference spectrum measured at a pump-probe delay of 75 ps as shown in Figure 2 of the main text, lies on a large O K-edge background signature. Figure S1a shows the raw pump-probe signature for an extended energy range of 530 to 620 eV. The spectrum in Figure S1a represents a single scan. Zooming into the Cr L-edge in Figure S1b, it becomes apparent that the O K-edge background signal is long-lived compared to the transient signal of $Cr(acac)_3$. We attribute the long-lived change as being due to heat dissipation to the solvent. The solvent contribution is removed either by fitting a polynomial to the 10 ns background or directly subtracting the 10 ns spectrum. Although polynomial subtraction is used in the main text, both methods give nearly identical results as this does not add noise from the background measurement to the final spectrum.

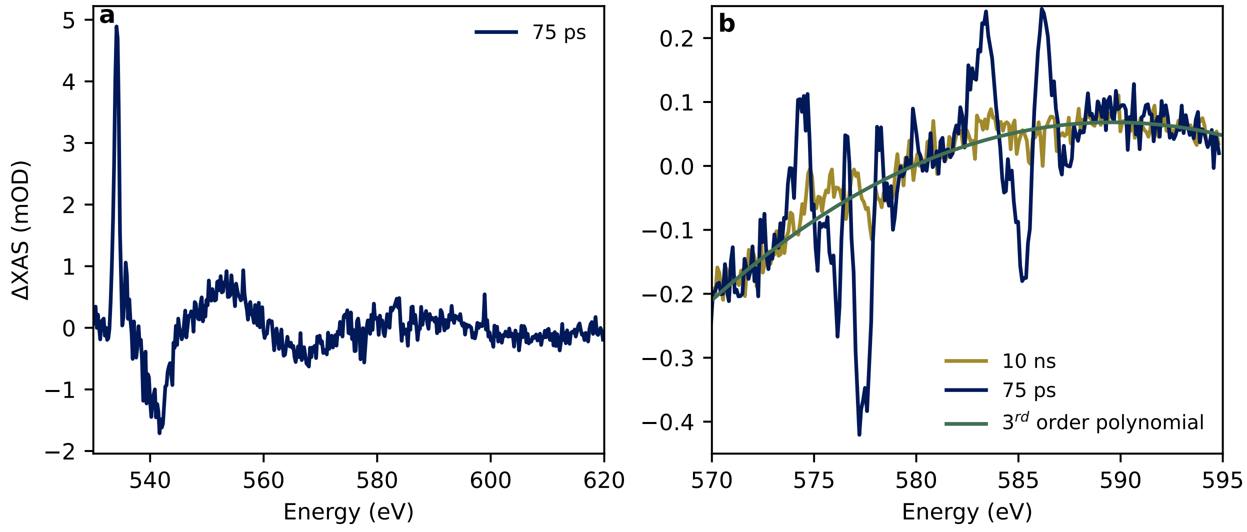

Figure S1: Transient O K-edge background subtraction. (a) A single scan of the spectral region of O K- and Cr $L_{2,3}$-edges, collected at 75 ps delay. (b) Comparison of the Cr L-edge spectra at 75 ps (blue) and 10 ns (gold), along with a polynomial fit (green) to the 10 ns background.

Kinetic Fit

The time trace shown in Figure S2 was measured at 577.6 eV and displays a negative transient signal that forms within the instrument's time resolution and decays to ∼50% of its maximum amplitude within ∼1 ns. This delay trace was fitted with the sum of a single exponential decay and a step function. Both functions were convoluted with a Gaussian instrument response function. The step function was included to represent the quasi-static solvent contribution, and its amplitude was fixed to 0.024 mOD to match the 10 ns solvent background amplitude at 577.6 eV (see Figure S1). The total fit is shown in black in Figure S2 with the individual components depicted as dashed red lines. The fitted time constant was found to be 1.26 ns, and the FWHM of the instrument response function, also used as a fitting parameter, was determined to be 85.5 ps, consistent with the expected time resolution.

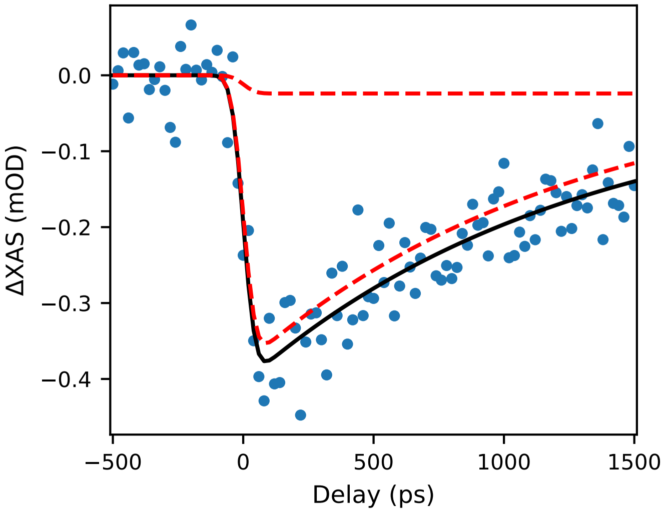

Figure S2: Kinetic fit of the delay trace measured at the maximum of the ground state depletion. The experimental data (blue scatter) are fitted with the sum of a step function and an exponential decay. The total fit is shown in black, with the individual components represented as red dashed lines.

Solvent Composition

The liquid jet running with pure EtOH and 15-20 mM Cr(acac)$_3$ exhibited large fluctuations. While the differential time-resolved signature can be measured, the static spectrum is more challenging under such conditions. Therefore, the solvent was then modified by the addition of 10% DMSO to increase solvent viscosity. This mixture led to a substantial stabilization of the liquid flat jet. Figure S3 shows that within the experimental noise, no significant differences were observed in the transient spectra for samples with and without the DMSO fraction. The data were merged to generate the final spectra shown in the main text.

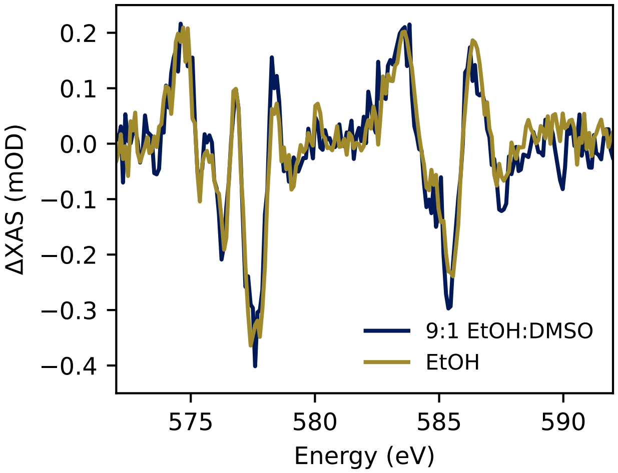

Figure S3: Transient X-ray absorption spectrum measured at 75 ps following laser excitation for both pure ethanol and a 1:9 DMSO:EtOH mixture.

Excited State Fraction

The spectrum of the ^{2}E state in Figure 4 of the main text was constructed from the ground state spectrum and the difference spectrum was measured at 75 ps following photoexcitation according to $S(^2E) = S(^4A_2) + \Delta A/f$ where f is the excited state fraction. It is not possible to determine the excited state fraction directly from the transient XAS measurement because it is a product of the fraction of sample that is initially excited and the quantum yield (QY) of the ^{2}E state at 75 ps delay. The measurements lack the time-resolution to observe the degree of initial excitation. While this fraction can in principle be estimated from the absorption

cross section, the experiment was carried out in the high-fluence limit where the absorption has saturated. Instead, we simply compare how the ^{2}E spectrum changes as a function of the excitation fraction.

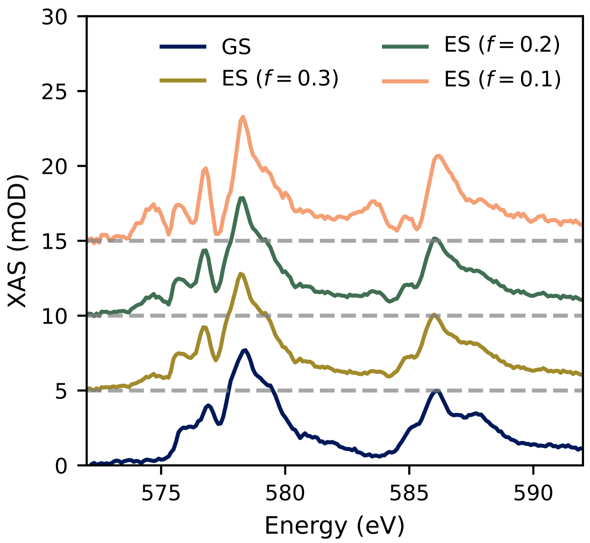

Figure S4: Excited state spectrum (ES) at 75 ps delay as a function of the excited state fraction. For comparison, the $^4A_2$ spectrum is shown (GS).

Figure S4 shows the excited state (ES) spectrum at 75 ps delay generated using 10, 20, and 30% excited state fractions. The ground state (GS) spectrum is shown for comparison. Regardless of the chosen fraction, the features denoted in the main text appear at the same energies. For example, the maximum of the B′ shifts less than 0.05 eV for excited state fractions between 0.1 and 0.4. Below 10% the spectrum exhibits unphysical negative-going features, and the 10% spectrum shown in Figure S4 has a nearly 0 mOD amplitude at 577.4, 584.5, and 585.2, which would be atypical for an L-edge XAS spectrum after the onset of absorption. The ^{2}E spectra generated with 20 and 30% excitation fractions yield much more reasonable spectra that exhibit only small differences.

Choosing a excitation fraction of greater than 10% implies that either/both a high fraction of the sample is initially excited, or/and there is a greater QY of the ^{2}E state than previously reported. The transient IR absorption measurements by Maçôas *et al.* on $Cr(acac)_3$ performed in tetrachloroethylene found a 17% QY when excited by 345 nm.[S1] If such a QY

still applied to the results reported here, this would imply a nearly 100% of the sample is initially excited to give a 20% yield. As with the ^{2}E lifetime discussed in the main text, sample temperature will affect the doublet yield. We have integrated the previously reported kinetic model for temperatures from 25 to -20 °C, and found that the QY increases from 19% to 34% over this temperature range. Based on this model, we note that an excitation fraction at 75 ps of 30% or greater is unlikely because this would require both a large fraction of the sample to be excited (∼90%) and a low temperature (< -10 °C). A 20% excited state fraction at 75 ps could be achieved by a temperature of liquid jet temperature of ∼0 °C (QY = 27.5%) and an initial excitation yield of ∼70%.

Theoretical Methods

Multiplet Simulations

All parameters for the simulations are given in Table S1.

Table S1: Parameters used for LFM Simulations.

Parameter	Value (eV)
Δ_0	2.32
F^2_{dd}	5.99
F^4_{dd}	5.31
F^2_{pd}	4.89
G^1_{pd}	3.59
G^3_{pd}	2.04
ζ_{2p}	5.668
ζ_{3d}	0.035

To compare the spin state contributions in the multiplet spectra with those from the *ab initio* results a method of determining spin fractions must be chosen. For this purpose, we use an approach similar to that of Zhang *et al.*[S2] where the final states computed in intermediate coupling are expressed as linear combinations of basis states that are eigenfunctions of the SOC-free Hamiltonian.

$$\hat{H}_{SF} = \hat{H}_{ee} + \hat{H}_{CF} \tag{1}$$

The eigenfunctions of this Hamiltonian are also eigenfunctions of $\hat{S}^2$ and can be grouped as doublets, quartets, and sextets. The re-expansion of the intermediate coupling states in the eigenstates of $\hat{H}_{SF}$ yields the weight of each multiplicity.

Quantum Chemistry Calculations

The calculations carried out in this work utilized either orbitals optimized at the CASSCF level of theory or the quasi-restricted orbitals (QROs) from a ground state DFT calculation. The CASSCF orbitals were generated from a state-averaged CASSCF(3,5) calculation including all doublet (40 roots) and quartet (10 roots). The QROs are generated using the functionals BP86,[S3,S4] TPSSh,[S5,S6] B3LYP,[S7] and BHandHLYP[S7] with Hartree-Fock (HF) exchange fractions of 0, 10, 20, and 50%, respectively. The active orbitals from a CASSCF(3,5) calculation and from TPSSh-QROs are shown on the left and right of Figure S5, respectively. While the differences are subtle it can be seen that the DFT orbitals are more delocalized onto the acac ligands than their CASSCF counterparts.

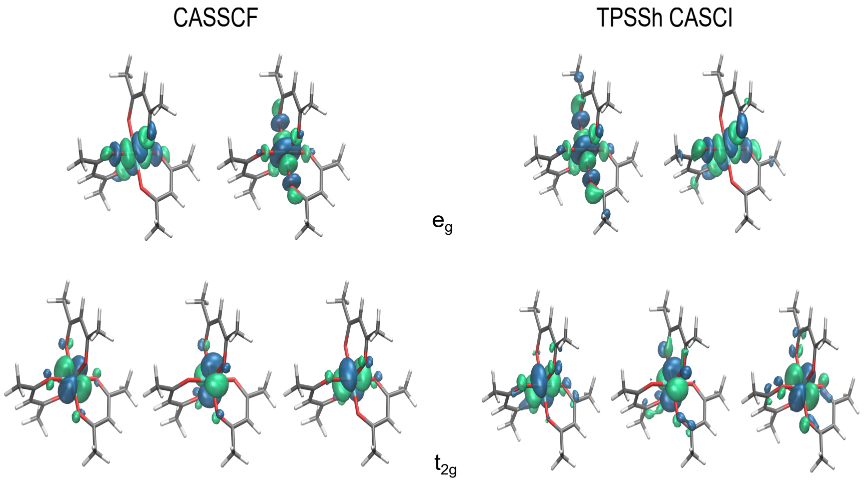

Figure S5: Molecular orbitals included in the active space of the CASSCF (left) and CASCI-TPSSh (right) calculations.

The spin and orbital analysis of the 2p XAS spectra were computed from state contributions to each excitation. Here we refer to the states resulting from the spin-free CASCI calculations as the SOC-free states and those resulting from including SOC as the SOC states. All transitions are between SOC states. Firstly, the spin contributions to spectra shown in Figures 4 and 5 of the main text were computed for each final state. The spin weights for each SOC state are given as the sum of the weights of the contributing SOC-free states for each spin multiplicity. The spectra for each multiplicity were generated by weighting the oscillator strength by the final state spin fraction. SOC-free states with weights $\geq 10^{-4}$ were considered. The same procedure generated the oscillator strength difference spectra comparing the $^4A_2$ and ^2E. The orbital difference spectra were computed similarly to the approach described by Pinjari *et al.*[S8] For each SOC state, the orbital occupation averages are computed as the average occupation of each SOC-free state. The differences are then taken between these numbers and the valence state occupation values. The orbital occupation averages for each SOC-free state are first computed considering all configurations that contribute with a weight $\geq 10^{-5}$. The difference spectra are given by multiplying the oscillator strength by the population differences for each transition and then convoluting the resulting stick with a Voigt profile for the total spectrum generation.

Computational Results

Ligand-Field Multiplet Simulations

The spin multiplicity contributions to the multiplet simulations were computed to further establish the similarity between the LFT spectrum and the CASCI-TPSSh result shown in Figure 3 of the main text. Figure S6 shows the spin decomposition of the LFT spectrum based on the procedure described above. For the ground state spectrum shown in Figure S6a, the overall lineshape follows that of the quartet component as it does with the CASCI-TPSSh approach. There are some subtle differences between the two methods. The doublet

weight is shifted slightly to higher energy in the case of the quantum chemical approach, and both the doublet and sextets more of a contribution to the lowest energy feature in the LFT case. For the valence excited state in Figure S6b, the result is nearly identical to the CASCI-TPSSh result where L_3 spectrum is comprised of a near-equal weighting of doublet and quartet peaks.

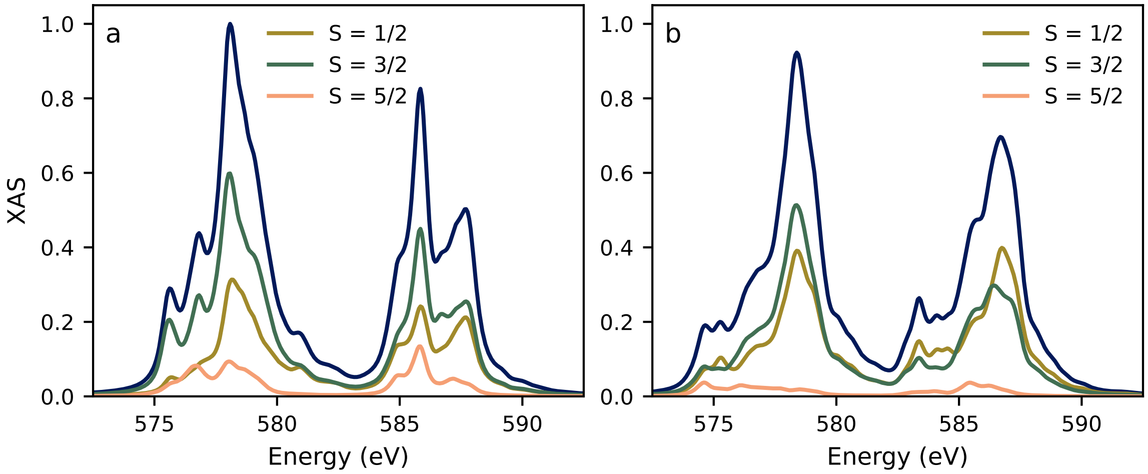

Figure S6: Spin state decomposition of ligand field multiplet simulations for the (a) $^4A_2$ and (b) ^2E states.

Quantum Chemical Calculations

Valence Excited States

The energies of the low-lying valence excited states of $Cr(acac)_3$ are tabulated in Table S2. The excitation energies are given for SA-CASSCF with and without NEVPT2 correlation energies for two active spaces. Firstly, the (3,5) CASSCF calculation includes just the five 3d orbitals filled by the 3 d-electrons (orbitals shown above). The CASSCF(13,13) calculation additionally includes 10 electrons coming from 2 σ bonding orbitals (4 electrons) and the 3 acac π HOMO orbitals (6 electrons). The three π^* acac LUMO orbitals are also included. The LFT values exhibit the best agreement with the published optical data. Inclusion of electron correlation via NEVPT2 greatly improves the accuracy of all of the CASSCF calculations. However, all quantum chemical methods significantly overestimate the $^4A_2 \rightarrow$

Table S2: Calculated vertical valence excitation energies for electronic states in D_3 symmetry with the O_h parent term given in parenthesis.

Transition	Method	Energy (eV)	Energy (cm^{-1})
$^4A_2 \rightarrow {}^2E$	LFT	1.74	14,055
	CASSCF(3,5)	2.38	19,167
	CASSCF(3,5) + NEVPT2	2.14	17,260
	CASSCF(13,13)	2.42	19,512
	CASSCF(13,13) + NEVPT2	2.05	16,527
	CASCI-TPSSh	2.05	16,516
$^4A_2 \rightarrow {}^2A$ ($^2T_1$)	LFT	1.76	14,465
	CASSCF(3,5)	2.48	20,012
	CASSCF(3,5) + NEVPT2	2.24	18,066
	CASSCF(13,13)	2.50	20,188
	CASSCF(13,13) + NEVPT2	2.10	16,980
	CASCI-TPSSh	2.11	16,998
$^4A_2 \rightarrow {}^2E$ ($^2T_1$)	LFT	1.76	14,402
	CASSCF(3,5)	2.51	20,267
	CASSCF(3,5) + NEVPT2	2.27	18,350
	CASSCF(13,13)	2.55	20,517
	CASSCF(13,13) + NEVPT2	2.16	17,440
	CASCI-TPSSh	2.18	17,528
$^4A_2 \rightarrow {}^4A$ ($^4T_2$)	LFT	2.32	18,712
	CASSCF(3,5)	2.02	16,310
	CASSCF(3,5) + NEVPT2	2.38	19,210
	CASSCF(13,13)	2.21	17,840
	CASSCF(13,13) + NEVPT2	2.47	19,894
	CASCI-TPSSh	1.95	15,680
$^4A_2 \rightarrow {}^4E$ ($^4T_2$)	LFT	2.32	18,712
	CASSCF(3,5)	2.05	16,575
	CASSCF(3,5) + NEVPT2	2.43	19,594
	CASSCF(13,13)	2.25	18,140
	CASSCF(13,13) + NEVPT2	2.53	20,350
	CASCI-TPSSh	1.96	15,806

^2E. The CAS(13,13)+NEVPT2 gives a value of 2.05 compared to the experimental value of ∼1.6 eV, which still possesses an error of more than 0.4 eV. The challenge of calculating accurate spin-flip energies in Cr(III) complexes is well-known.[S9,S10] The *ab initio* energies for the states arising from the $^4T_2$ are much closer to the experimentally determined values. Finally, the energies from the CASCI-TPSSh calculation are given. Surprisingly, the energies of the doublet excited states from the CASCI+TPSSh calculation are in excellent agreement with CASSCF(13,13)+NEVPT2 values, but the quartet excitation energy is greatly underestimated in the case of the DFT orbitals.

Functional Dependence of CASCI Spectra

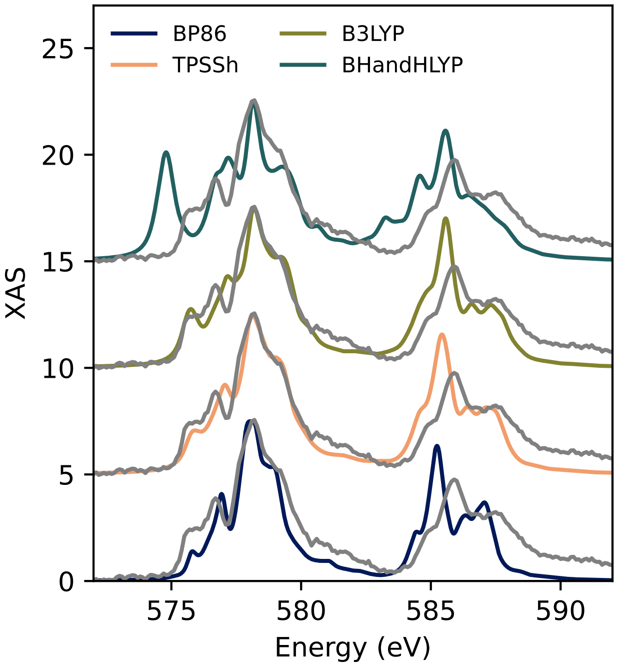

Figure S7: Comparison of calculated CASCI spectra (color) for wavefunctions employing DFT QROs and experiment (gray) for the $^4A_2$ ground state of $Cr(acac)_3$. Each calculated spectrum is energetically shifted to align it with the maximum of the experimental spectrum. The intensity is similarly scaled to the OD of the experimental spectrum.

Figures S7 and S8 show the comparison of CASCI-DFT and experimental XAS spectrum for the $^4A_2$ ground state and the ^2E excited states, respectively. The XAS spectrum has been calculated using orbitals from four different density functionals chosen to give a range

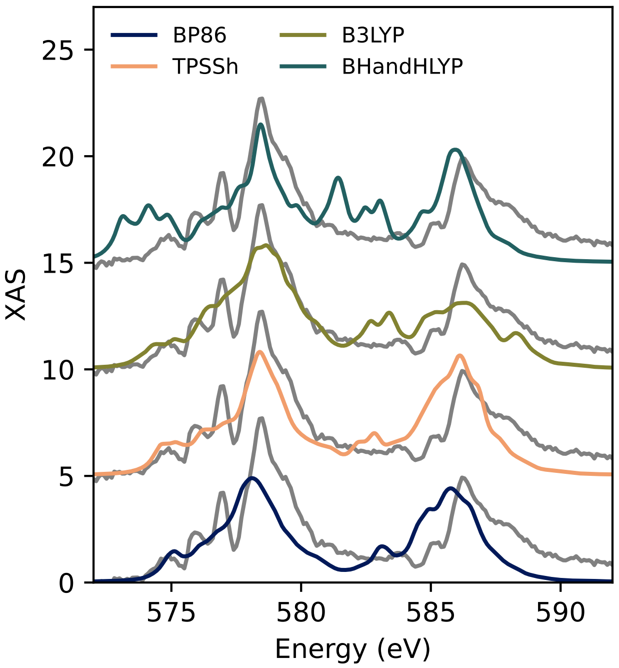

Figure S8: Comparison of calculated CASCI spectra (color) for wavefunctions employing DFT QROs and experiment (gray) for the ^2E excited state of $Cr(acac)_3$. Each calculated spectrum is shifted by the same amount as the ground state spectrum and the intensity is scaled by the same value as the ground state.

of Hartree-Fock (HF) exchange fractions. The rationale is that the CASSCF orbitals are often found to be too ionic. By using DFT orbitals the metal-ligand covalency can be tuned by adjusting the fraction of HF exchange. The variation in d-character in the active space 3d orbitals can be in Table S3. The computed spectra show that the spread of the multiplets grows significantly when going from BP86 to BHandHLYP. TPSSh with 10% HF exchange fraction gives the best agreement with the experiment. B3LYP also gives a reasonable ground state spectrum, but the agreement worsens for the excited state. BP86 underestimates the width of the L_3-edge, particularly for the excited state, and the spectrum given by BHandHLYP is too broad and too structured for both ground and excited state.

Comparison of CASCI and CASCI+NEVPT2

Here we report the results of the purely *ab initio* CASCI calculations that utilize orbitals from a state-averaged CASSCF(3,5) calculation. Comparison of experiment with the CASCI

Table S3: Percent of d-orbital character for active valence MOs given by the average Löwdin orbital populations in approximate O_h symmetry.

Method	t_{2g}	e_g
BP86	87	73
TPSSh	91	75
B3LYP	92	76
BHandHLYP	95	78
CASSCF(3,5)	96	88

spectra with an without NEVPT2 correction are is shown in Figure S9. For the $^4A_2$ ground state, neither the CASCI nor the CASCI+NEVPT2 calculations successfully recover the double pre-peak (labeled A and B in the main text). Furthermore, the intensity of the shoulder observed on the high-energy side of the main feature, which occurs at approximately 579.5 eV, is significantly overestimated in the calculations. In the case of the ^2E excited state, the CASCI result exhibits good agreement with the experiment, and state analysis yields the same interpretation as the CASCI-TPSSh calculation. However, inclusion of NEVPT2 for the ^2E results in a spectrum with a strongly red-shifted pre-edge and significantly worse agreement with the experimental spectrum than for any other method.

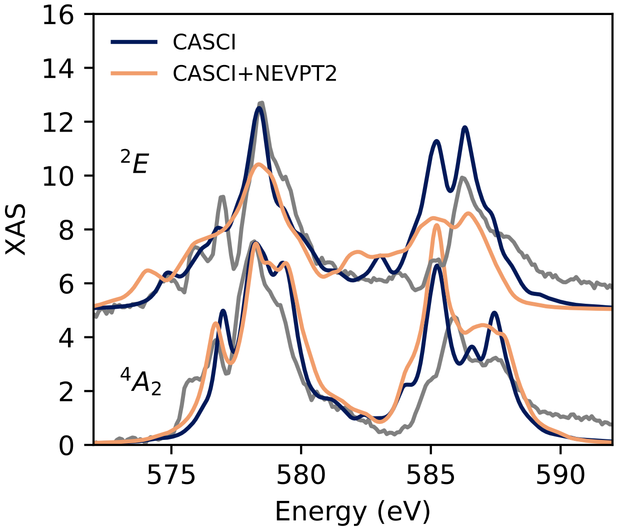

Figure S9: Comparison of CASCI L_3-edge XAS spectrum of $Cr(acac)_3$ calculated at both the CASCI-CASSCF(3,5) (dark blue) and at the CASCI-CASSCF(3,5)+NEVPT2 (orange) levels of theory. Spectra for the ^2E excited state and the $^4A_2$ ground state are given on top and bottom, respectively. Experimental spectra are shown in gray.

References

(S1) Maçôas, E. M. S.; Mustalahti, S.; Myllyperkiö, P.; Kunttu, H.; Pettersson, M. Role of Vibrational Dynamics in Electronic Relaxation of Cr(acac)$_3$. *J. Phys. Chem. A* **2015**, *119*, 2727–2734.

(S2) Zhang, K.; Girolami, G. S.; Vura-Weis, J. Improved charge transfer multiplet method to simulate *M* - and *L* -edge X-ray absorption spectra of metal-centered excited states. *J. Synchrotron Radiat.* **2018**, *25*, 1600–1608.

(S3) Becke, A. D. Density-functional exchange-energy approximation with correct asymptotic behavior. *Phys. Rev. A* **1988**, *38*, 3098–3100.

(S4) Perdew, J. Density-functional approximation for the correlation energy of the inhomogeneous electron gas. *Phys. Rev. B* **1986**, *33*, 8822–8824.

(S5) Tao, J.; Perdew, J. P.; Staroverov, V. N.; Scuseria, G. E. Climbing the Density Functional Ladder: Nonempirical Meta–Generalized Gradient Approximation Designed for Molecules and Solids. *Phys. Rev. Lett.* **2003**, *91*, 146401.

(S6) Staroverov, V. N.; Scuseria, G. E.; Tao, J.; Perdew, J. P. Comparative assessment of a new nonempirical density functional: Molecules and hydrogen-bonded complexes. *J. Chem. Phys.* **2003**, *119*, 12129–12137.

(S7) Becke, A. D. A new mixing of Hartree–Fock and local density-functional theories. *J. Chem. Phys.* **1993**, *98*, 1372–1377.

(S8) Pinjari, R. V.; Delcey, M. G.; Guo, M.; Odelius, M.; Lundberg, M. Restricted active space calculations of L-edge X-ray absorption spectra: From molecular orbitals to multiplet states. *J. Chem. Phys.* **2014**, *141*, 124116.

(S9) Ribbing, C.; Pierloot, K.; Ceulemans, A. Ab Initio Calculations of the Trigonal and

Zero-Field Splittings in Trischelated Diketonato Complexes of Trivalent Chromium. *Inorg. Chem.* **1998**, *37*, 5227–5232.

(S10) Atanasov, M.; Andreici Eftimie, E.-L.; Avram, N. M.; Brik, M. G.; Neese, F. First-Principles Study of Optical Absorption Energies, Ligand Field and Spin-Hamiltonian Parameters of Cr^{3+} Ions in Emeralds. *Inorg. Chem.* **2022**, *61*, 178–192.

xyz coordinates

Coordinates in angstrom (Å) of $Cr(acac)_3$ from BP86/def2-TZVP geometry optimization.

```
Cr     -0.066161     -0.093813     -0.014851
O      -0.090610     -2.060996     -0.121227
O      -2.034575     -0.010543      0.015035
O      -0.096161     -0.012921     -1.983109
O      -0.003650      1.871551      0.126595
O       1.903835     -0.136340     -0.074093
O      -0.069403     -0.203278      1.951856
C      -1.131686     -2.817966     -0.118023
C      -2.458669     -2.359717     -0.061893
C      -2.845574     -1.010223     -0.004285
C      -0.849771     -4.294798     -0.181717
C      -4.306351     -0.651116      0.036483
C      -0.056013      1.049136     -2.709077
C       0.005768      2.361452     -2.211019
C       0.026303      2.709800     -0.849971
C      -0.083621      0.810865     -4.194647
C       0.087049      4.158993     -0.449304
C       2.700727     -0.128369      0.936796
C       2.294732     -0.143085      2.281930
C       0.961490     -0.188879      2.722693
C       4.166479     -0.104850      0.596911
C       0.659426     -0.231712      4.196252
H      -3.248791     -3.107913     -0.069087
H      -0.265418     -4.516229     -1.087245
H      -1.769165     -4.890516     -0.186568
H      -0.231853     -4.584745      0.681371
H      -4.551381     -0.024837     -0.834545
H      -4.509777     -0.049946      0.935115
H      -4.949054     -1.538353      0.037490
```

H	0.035601	3.172423	-2.936054
H	-1.014552	0.288439	-4.462106
H	0.750379	0.150179	-4.474108
H	-0.016496	1.744784	-4.763381
H	0.987207	4.331115	0.159662
H	-0.781644	4.399766	0.181518
H	0.102685	4.826301	-1.318004
H	3.074188	-0.134567	3.041172
H	4.410747	-0.977931	-0.026431
H	4.797653	-0.108693	1.492387
H	4.386724	0.792023	-0.001331
H	0.098831	-1.150139	4.426707
H	0.012001	0.617685	4.460399
H	1.569649	-0.199902	4.805128
